# The Effects of Curcumin on Weight Loss Among Patients With Metabolic Syndrome and Related Disorders: A Systematic Review and Meta-Analysis of Randomized Controlled Trials

**DOI:** 10.3389/fphar.2019.00649

**Published:** 2019-06-12

**Authors:** Maryam Akbari, Kamran B. Lankarani, Reza Tabrizi, Majid Ghayour-Mobarhan, Payam Peymani, Gordon Ferns, Amir Ghaderi, Zatollah Asemi

**Affiliations:** ^1^Health Policy Research Center, Institute of Health, Student Research Committee, Shiraz University of Medical Sciences, Shiraz, Iran; ^2^Health Policy Research Center, Shiraz University of Medical Sciences, Shiraz, Iran; ^3^Metabolic Syndrome Research Center, School of Medicine, Mashhad University of Medical Sciences, Mashhad, Iran; ^4^Division of Medical Education, Brighton and Sussex Medical School, Brighton, United Kingdom; ^5^Department of Addiction Studies, School of Medicine, Kashan University of Medical Sciences, Kashan, Iran; ^6^Clinical Research Development Unit-Matini/Kargarnejad Hospital, Kashan University of Medical Sciences, Kashan, Iran; ^7^Research Center for Biochemistry and Nutrition in Metabolic Diseases, Kashan University of Medical Sciences, Kashan, Iran

**Keywords:** curcumin, weight loss, meta-analysis, leptin, BMI

## Abstract

**Background and objective:** The current systematic review and meta-analysis of randomized controlled trials (RCTs) was carried out to assess the influence of curcumin intake on weight among patients with metabolic syndrome and related disorders.

**Methods:** We searched the following databases up until January 2018: MEDLINE, EMBASE, Web of Science, and Cochrane Central Register of Controlled Trials. The relevant data were extracted and evaluated for quality of the studies in accordance with the Cochrane risk of bias tool. Data were pooled using the inverse variance method and expressed as standardized mean difference (SMD) with 95% confidence intervals (95% CI).

**Results:** Eighteen articles (21 studies) that comprised a total of 1,604 individuals were finally included in the meta-analysis. Curcumin intake significantly reduced body mass index (BMI) (SMD −0.37; 95% CI, −0.61, −0.13; *P* < 0.01), weight (SMD −0.23; 95% CI, −0.39, −0.06; *P* < 0.01), waist-circumference (WC) (SMD −0.25; 95% CI, −0.44, −0.05; *P* = 0.01), leptin levels (SMD −0.97; 95% CI, −1.18, −0.75; *P* < 0.001) and increased adiponectin levels (SMD 1.05; 95% CI, 0.23, 1.87; *P* = 0.01). We found no significant effect of curcumin intake on hip ratio (HR) (SMD −0.17; 95% CI, −0.42, 0.08; *P* = 0.18).

**Conclusions:** Overall, we have found that curcumin intake among patients with metabolic syndrome and related disorders was correlated with a significant reduction in BMI, weight, WC, and leptin, and a significant increase in adiponectin levels, but did not affect HR.

## Introduction

Metabolic syndrome (MetS), obesity, and type 2 diabetes mellitus (T2DM) are rapidly increasing in prevalence globally, possibly related to poor lifestyle habits, a high calorie diet, low physical activity, and smoking (Ewang-Emukowhate et al., 2014). MetS is defined as the concurrence of obesity-associated cardiovascular risk factors, such as hyperinsulinemia, abdominal obesity, hypertriglyceridemia, reduced high-density lipoprotein (HDL)-cholesterol levels, and/or hypertension (Tune et al., 2017). In parallel with the obesity pandemic, MetS is an increasing in prevalence, and affects approximately 20% of people in the world (Ofori-Asenso et al., 2017). Both MetS and overweight are associated with elevated risk of mortality, metabolic disturbances, T2DM, vascular complications including atherosclerotic disease and coronary heart disease (CHD) (Phillips, 2013; Katsiki et al., 2014; Santilli et al., 2017).

There are some data that have shown that some spices, including curcumin may play a key function in the management of overweight individuals. Curcumin is a bioactive polyphenol component which is found in turmeric rhizomes and is commonly referred to as diferuloylmethane (Chainani-Wu, 2003). Clinical trials of curcumin on weight- and body mass index (BMI)-lowering effect have not been systematically evaluated, and the results are inconsistent. Di Pierro et al. (Di Pierro et al., 2015), have reported that a bioavailable form of curcumin resulted in improved weight management in overweight subjects. In addition, a significant decline in BMI and liver fat status was observed after the intake of 70 mg/day curcumin for 8 weeks among people with non-alcoholic fatty liver disease (NAFLD) (Rahmani et al., 2016). Some other studies have also documented a significant effect of curcumin intake on indicators of body composition (Chuengsamarn et al., 2012; Panahi et al., 2017a). However, anthropometric parameters such as BMI, weight, waist and hip circumference, total body fat, and parameters of oxidative stress were not affected following the intake of curcumin at a dosage of 1 g/day in obese people (Mohammadi et al., 2013) and 1 g/day in people with MetS (Ghazimoradi et al., 2017). Moreover, another study reported no significant effect of curcumin intake on weight or BMI (Yang et al., 2014).

It has been suggested that curcumin intake may improve weight and metabolic status through increasing basal metabolic rate, which may in turn cause increased energy expenditure (Bercik et al., 2010). We are aware of no systematic review and meta-analysis of randomized controlled trials (RCTs) on the effect of curcumin intake on weight loss in people with metabolic diseases. This meta-analysis was conducted to summarize the existing evidence of RCTs to evaluate the impact of curcumin intake on weight loss in patients with MetS and related disorders.

## Methods

### Search Strategy and Selection Studies

RCTs were searched systematically using several online databases: The Cochrane Library, EMBASE, PubMed, and Web of Science databases up until January 2018. For all ongoing trials, we conducted searches of two reference databases—Databases of International Standard Randomized Controlled Trial Number Register and Meta-register for RCTs. RCTs retrieved that have investigated the association between curcumin intake and body composition using the following Medical Subject Headings (MeSH) and search terms: patients (“obese” OR “overweight” OR “T2DM” OR “hyperlipidemic” OR “NAFLD” OR “MetS”), intervention (“curcumin” OR “turmeric” OR “curcuminoids” AND “intake” OR “supplementation”), and outcomes (“body mass index [BMI]” OR “body weight” OR “waist-circumference [WC]” OR “hip ratio [HR]”OR “leptin” OR “adiponectin”). Two authors (MA and ZA) independently conducted a systematic search from databases. Reference lists of selected studies and previous review article were manually reviewed for additional relevant articles. This study was restricted to RCTs published in the English language.

### Eligibility Criteria

RCTs were selected using a two-stage process by two researchers (MA and RT), independently. In the first stage, researchers screened the titles and/or abstracts to include RCTs had eligibility into our meta-analysis. In the second, the full texts of eligibility RCTs were retrieved to evaluate with more details. The eligibility criteria to include into our meta-analysis were human and original trials, and the administration of curcumin supplements in intervention group, whereas placebo in the comparison group. RCTs that did not report the mean changes or mean difference of body composition, serum leptin, and adiponectin along with standard deviation (SD) for the treatment and control groups, case reports, the abstracts of congress without full text; and RCTs that did not achieve at least required score of quality assessment, were excluded.

### Data Extraction and Quality Assessment

Data extraction and quality assessment of RCTs were carried out by two independent authors (ZA and MA), according to standard forms in Excel software and the Cochrane Collaboration risk of bias tool, respectively. These forms were contained the following data: author’s name, publication year, country where the study was conducted, study design, dosage of curcumin supplements (mg/day), the intervention and placebo type, the duration of treatment, sample size (intervention and placebo), the mean and standard deviation of the body composition, serum leptin, and adiponectin. The Cochrane Collaboration risk of bias tool includes the following domains related to quality of RCTs: random sequence generation, allocation concealment, blinding process and outcome assessment, and withdrawal of patients. In the case of discrepancy, resolved by discussion with third author (ZA).

### Data Analysis

RevMan V.5.3 software (Cochrane Collaboration, Oxford, UK) and Stata version 12.0 (Stata Corp., College Station, TX) were used for statistical analyses. Heterogeneity was assessed using the Cochran’s *Q* test and *I*-square statistic. *I*-square higher than 50% with *P*-value <0.05 indicated the existing heterogeneity. The inverse variance method and Cohen statistics were used for calculation of standardized mean difference (SMD) with 95% confidence interval (CI). In this study, because of different indications among the included RCTs were conducted a random-effects model for meta-analysis was used to establish the relation between curcumin intake and body composition, leptin, and adiponectin levels. However, subgroup analyses were performed to evaluate the source of heterogeneity based on suspected variables such as dosage of curcumin (≤500 mg/day vs. >500 mg/day) and the duration of study (≤8 weeks vs. >8 weeks) between primary RCTs. In addition, sensitivity analyses were conducted in the trials one by one to assess the reliability of the pooled SMDs. The existence of potential publication bias was evaluated through using Egger’s tests for the primary outcome measure. *P*-values less than 0.05 were considered to be statistically significant.

## Results

**Figure 1:**
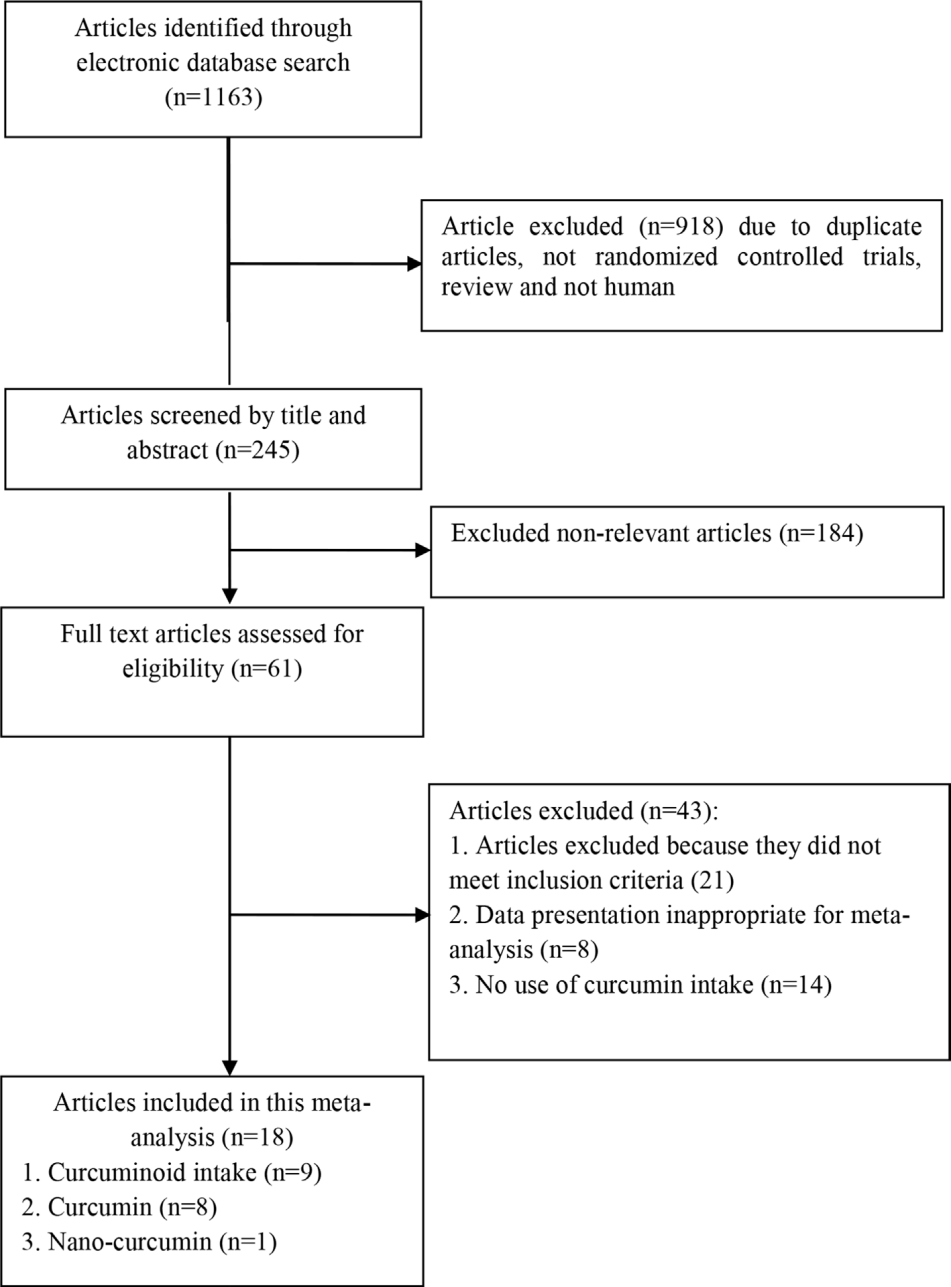
Literature search and review flowchart for selection of studies.

**Figure 2:**
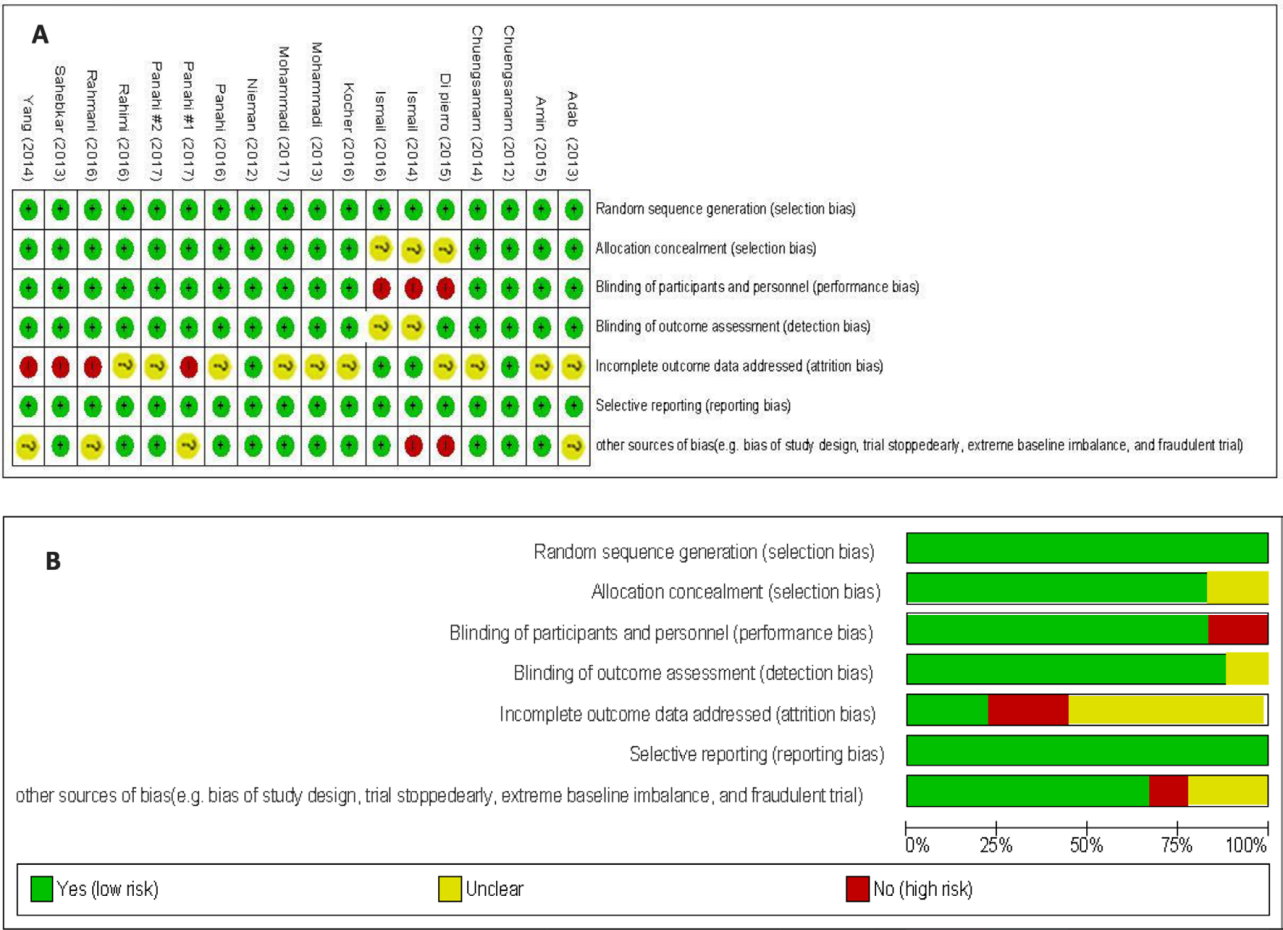
The methodological quality of included studies on effect of curcumin on body composition and adipokines based on review authors’ judgments about each risk of bias item presented as percentages across all included studies **(A)** and each risk of bias item for each included study **(B)**.

Administered curcumin in trials included in the present meta-analysis was as powder, or extract. A total of 1,163 studies were identified though our initial literatures search. After screening RCTs, 18 articles (21 trials) were determined to be appropriate for in the present meta-analysis. The flow diagram of the step-by-step study identification and selection is shown in **Figure 1**. Characteristics of RCTs for nine curcuminoid supplementation trials, eight curcumin supplementation trials, and one nano-curcumin trial included in this meta-analysis are presented in **Table 1**. RCTs were published from 2012 to 2017. In total, the meta-analysis was conducted on 1,646 participants with various total sample size from 25 to 234 persons. Fifteen articles were double-blind, and three studies were single-blind. 14 articles used parallel design, and four used crossover design. Fourteen trials have showed changes on BMI, 15 on body weight, 9 on WC, 5 on HR, 4 on leptin, and 5 on adiponectin. Total daily dose of curcumin intake or curcumin consumption varied between 70 and 2,400 mg/day. The duration of treatment among studies was between 4 and 36 weeks. The methodological quality of included trials into present meta-analysis was evaluated using the Cochrane Collaboration risk of bias tool based on authors’ judgments about each risk of bias item for each included study is shown in **Figure 2**.

**Table 1:** The characteristics of included randomized controlled trials (RCTs).

Authors (Ref)	Publication year	Country	Intervention/control (sample size)	Duration (week)	Dosage (mg/day)	Patients	Type of intervention	Mean age (control/intervention)
Adab et al. (2013)	2013	Iran	39/36	8	2,100 mg of turmeric powder daily	Hyperlipidemic patients with type 2 diabetes mellitus	Turmeric powder	55.66 ± 8.64, 54.76 ± 6.00
Amin et al. (2015)	2015	Pakistan	63/63	8	2.4 g/day (800 mg in three capsule)	Metabolic syndrome	Powdered rhizome of *Curcuma longa* (turmeric)	41.57 ± 12.8, 42.4 ± 13.7
Chuengsamarn et al. (2014)	2014	Thailand	107/106	24	Curcuminoid content of 250 mg (six capsules daily)	Type 2 diabetes mellitus	Curcumin capsule (rhizomes of turmeric)	59.58 ± 10.70, 59.16 ± 10.96
Chuengsamarn et al. (2012)	2012	Thailand	117/117	36	Curcuminoid content of 250 mg (six capsules daily)	Type 2 diabetes mellitus	Dried rhizomes of turmeric (*Curcuma longa* Linn).	57.93 ± 12.76, 56.95 ± 11.89
Di Pierro et al. (2015)	2015	Italy	22/22	8	800 mg/daily	Metabolic syndrome (overweight)	Curcumin-based product-*Curcuma longa* extract	41.85 ± 15.91, 39.10 ± 16.8
Ismail et al. (2016)	2016	Egypt	15/14	4	One capsule containing 500 mg curcumin	Pediatrics obese subjects	Curcuminoids are extracted from turmeric (*Curcuma longa* root)	14.7 ± 4.52
Ismail et al. (2016)	2016	Egypt	15/14	4	One capsule containing 500 mg curcumin	Adults obese subjects	Curcuminoids are extracted from turmeric (*Curcuma longa* root)	37.55 ± 9.93
Ismail et al. (2014)	2014	Egypt	14/11	4	One capsule containing 500 mg curcumin	Obese children	Curcuminoids are extracted from turmeric (*Curcuma longa* root)	15.57 ± 5.79, 16.53 ± 8.7
Kocher et al. (2016)	2016	Germany	42/42	6	294 mg curcuminoids per day (as micelles)	Hyperlipidemic individuals (overweight)	Curcumin powder	51.19 ± 17.61
Mohammadi et al. (2017)	2017	Iran	36/36	6	200 mg pure curcumin per day	Metabolic syndrome	Curcumin capsule	38.59 ± 10.28, 37.52 ± 9.47
Mohammadi et al. (2013)	2013	Iran	15/15	4	500 mg C3 capsules containing 500 mg (1,000 mg/day)	Obese patients	Curcuminoids plus 5 mg BioPerine	37.9 ± 12.7, 39.0 ± 9.0
Nieman et al. (2012)	2012	USA	30/30	4	TM (2.8 g/day) or ∼112 mg curcumin	Overweight females	Turmeric capsule	55.7 ± 1.4
Panahi et al. (2017b)	2017	Iran	44/43	8	500 mg C3 capsules containing 500 mg (1,000 mg/day)	Non-alcoholic fatty liver disease	Curcumin capsule (phytosomal form)	47.21 ± 10.29, 44.98 ± 12.59
Panahi et al. (2017a)	2017	Iran	50/50	8	500 mg C3 capsules containing 500 mg (1,000 mg/day)	Type 2 diabetes mellitus	Curcuminoids plus 5 mg bioperine	41 ± 7, 43 ± 8
Panahi et al. (2016)	2016	Iran	50/50	8	500 mg C3 capsules containing 500 mg (1,000 mg/day)	Metabolic syndrome	Curcuminoids plus 5 mg bioperine	43.46 ± 9.70, 44.80 ± 8.67
Rahimi et al. (2016)	2016	Iran	35/35	12	80 mg/day	Type 2 diabetes mellitus	Nano-curcumin	60.95 ± 10.77, 56.34 ± 11.17
Rahmani et al. (2016)	2016	Iran	37/40	8	500 mg/day	Non-alcoholic fatty liver disease	Curcumin powder	48.95 ± 9.78, 46.37 ± 11.57
Sahebkar et al. (2013)	2013	Iran	15/15	4	500 mg C3 capsules containing 500 mg (1,000 mg/day)	Obese individuals	Curcuminoids plus 5 mg bioperine	38.43 ± 10.84
Yang et al. (2014)	2014	Taiwan	30/29	12	Curcumin extract capsule (630 mg three times daily) turmeric (*C. longa*) extract	Metabolic syndrome (overweight)	Curcumin capsule	59.61 ± 14.09, 59.03 ± 10.10

TM, tumeric.

### Pooled Effects of Curcumin Supplementation on Body Composition

**Figure 3:**
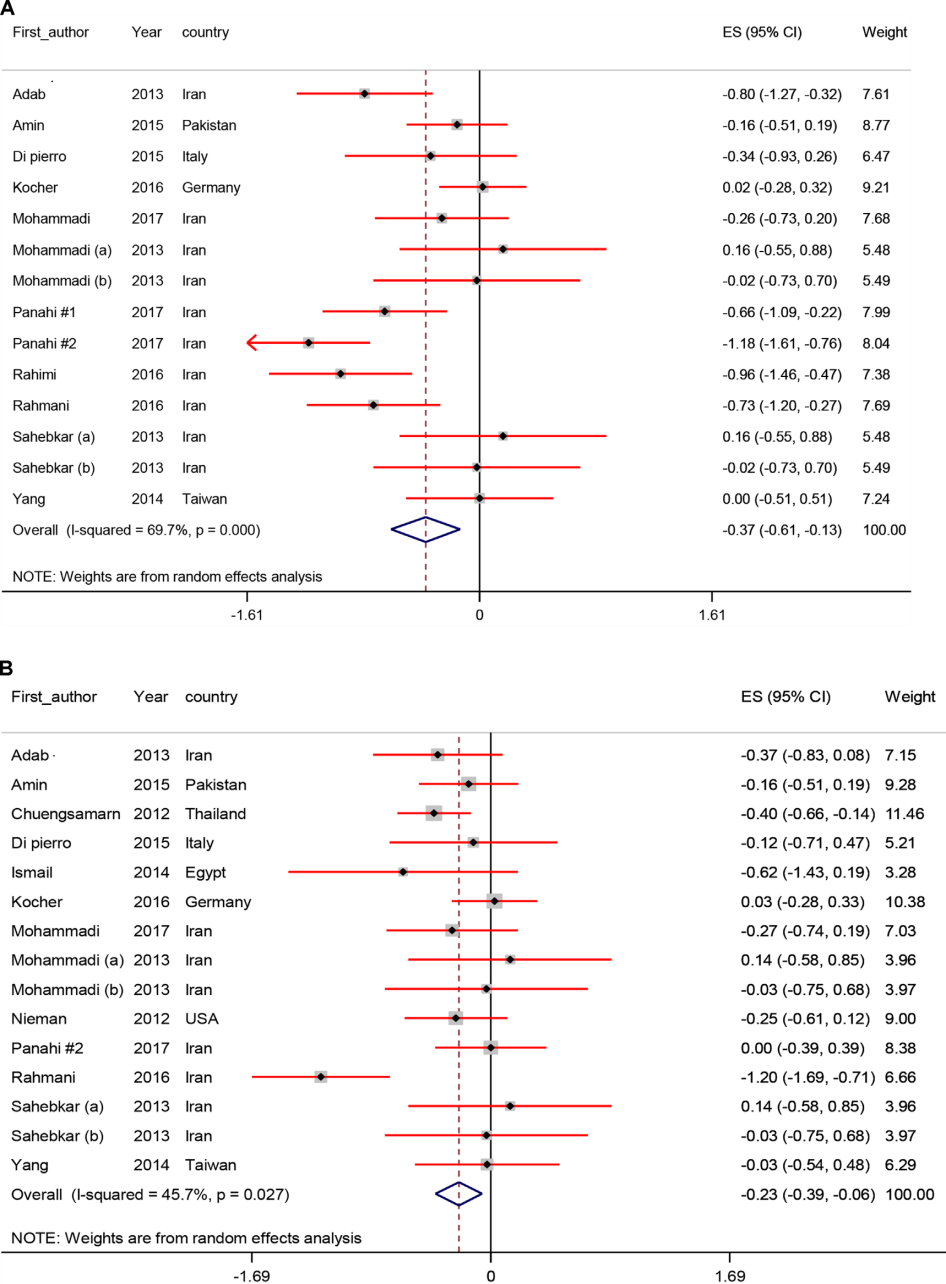

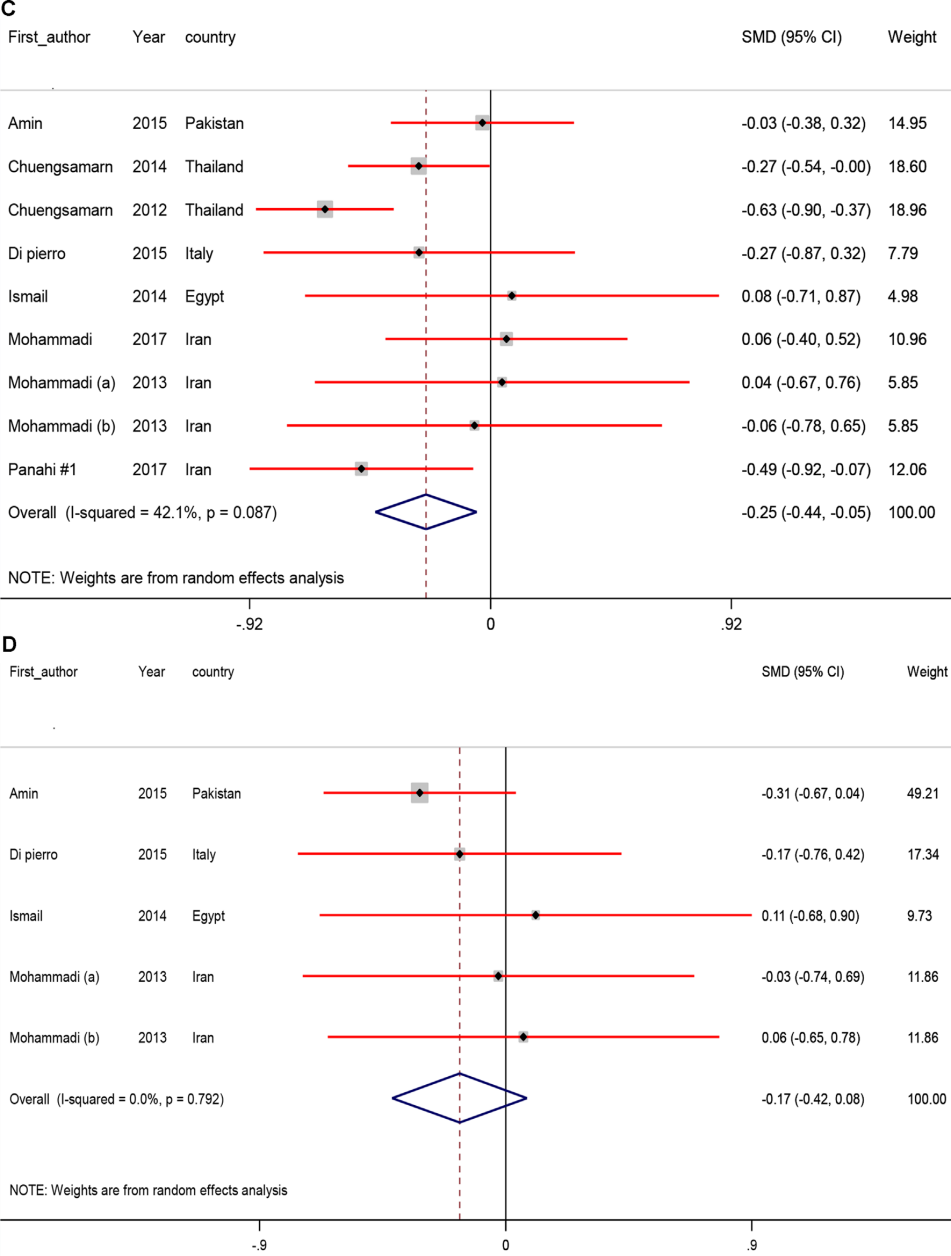
Meta-analysis body composition standardized mean difference estimates for **(A)** BMI, **(B)** for weight, **(C)** for waist-circumference (WC), and **(D)** for hip ratio (HR) in curcumin supplements and placebo groups (CI = 95%).

**Figure 3** shows the forest plots for the effect of curcumin supplementation on body composition. Curcumin resulted a significant reduction in BMI (SMD −0.37; 95% CI, −0.61, −0.13; *P* < 0.01), weight (SMD −0.23; 95% CI, −0.39, −0.06; *P* < 0.01), and WC levels (SMD −0.25; 95% CI, −0.44, −0.05; *P* = 0.01). We observed no significant effect of curcumin on HR (SMD −0.17; 95% CI, −0.42, 0.08; *P* = 0.18).

All meta-analyses of the study subject included RCTs with data before and after the intervention and control groups are presented in **Table 2**.

**Table 2:** The effects of curcumin intake on body mass index (BMI), weight, and body fat reduction based on subgroup analysis.

Variable			Number of study	Standardized mean difference	CI 95%	Heterogeneity		
						*I* ^2^ (%)	Q	*P*-value
Body composition	BMI	Intervention group (after vs. before)	14	−0.18	−0.31, −0.05	0.0	4.87	0.97
		Placebo group (after vs. before)	14	−0.01	−0.14, 0.12	0.0	1.55	0.99
		Change intervention group vs. placebo group	14	−0.37	−0.61, −0.13	69.7	42.95	<0.001
	Body weight	Intervention group (after vs. before)	15	−0.14	−0.26, −0.02	0.0	3.91	0.99
		Placebo group (after vs. before)	15	0.04	−0.08, 0.16	0.0	3.25	0.99
		Change intervention group vs. placebo group	15	−0.23	−0.39, −0.06	45.7	25.80	0.02
	WC	Intervention group (after vs. before)	9	−0.27	−0.41, −0.14	0.0	4.26	0.83
		Placebo group (after vs. before)	9	−0.00	−0.14, 0.13	0.0	6.50	0.59
		Change intervention group vs. placebo group	9	−0.25	−0.44, −0.05	42.1	13.82	0.08
	HR	Intervention group (after vs. before)	5	−0.24	−0.48, 0.01	0.0	0.49	0.97
		Placebo group (after vs. before)	5	−0.10	−0.34, 0.15	0.0	1.02	0.90
		Change intervention group vs. placebo group	5	−0.17	−0.42, 0.08	0.0	1.69	0.79
Adipokines	Leptin	Intervention group (after vs. before)	4	−1.36	−1.77, −0.95	58.9	7.29	0.06
		Placebo group (after vs. before)	4	−0.08	−0.28, 0.13	0.0	1.98	0.57
		Change intervention group vs. placebo group	4	−0.97	−1.18, −0.75	0.0	1.81	0.61
	Adiponectin	Intervention group (after vs. before)	5	1.22	0.17, 2.27	96.4	111.23	<0.001
		Placebo group (after vs. before)	5	0.23	−0.29, 0.75	87.7	32.46	<0.001
		Change intervention group vs. placebo group	5	1.05	0.23, 1.87	94.5	72.30	<0.001

WC, waist-circumference; HR, hip ratio.

Subgroup analyses for all body composition were conducted based on suspected variables for heterogeneity including dosage of curcumin and duration of study. However, the results of subgroup analyses showed that the reduction of heterogeneity was found in some of specific strata of suspected variables (**Table 3**).

**Table 3:** The effects of curcumin supplementation on body composition based on subgroup analysis.

Variable		Number of SMD included	Subgroups	Pooled effect estimate	95% CI	*I* ^2^ (%)	Overall *I* ^2^ (%)
BMI	Dosage of curcumin (mg/day)	4	≤500 (mg/day)	−0.46	−0.93, 0.01	79.2	69.7
		10	>500 (mg/day)	−0.33	−0.63, −0.03	68.2	
	Duration of study (week)	10	≤8 weeks	−0.42	−0.69, −0.14	72.1	
		4	>8 weeks	−0.24	−0.79, 0.32	71.1	
Body weight	Dosage of curcumin (mg/day)	5	≤500 (mg/day)	−0.43	−0.85, −0.00	78.1	45.7
		10	>500 (mg/day)	−0.19	−0.33, −0.04	0.0	
	Duration of study (week)	11	≤8 weeks	−0.25	−0.46, − 0.04	54.9	
		4	>8 weeks	−0.22	−0.47, 0.03	52.4	
WC	Dosage of curcumin (mg/day)	2	≤500 (mg/day)	0.07	−0.33, 0.46	0.0	42.1
		7	>500 (mg/day)	−0.31	−0.51, −0.10	41.4	
	Duration of study (week)	5	≤8 weeks	−0.15	−0.36, 0.07	5.1	
		4	>8 weeks	−0.34	−0.64, −0.04	51.6	
HR	Dosage of curcumin (mg/day)	1	≤500 (mg/day)	0.11	−0.68, 0.90	-	0.0
		4	>500 (mg/day)	−0.20	−0.46, 0.06	0.0	
	Duration of study (week)	3	≤8 weeks	−0.23	−0.51, 0.06	0.0	
		2	>8 weeks	0.02	−0.49, 0.53	0.0	
Leptin	Dosage of curcumin (mg/day)	2	≤500 (mg/day)	−0.86	−1.42, −0.30	6.0	0.0
		2	>500 (mg/day)	−0.99	−1.22, −0.75	0.0	
	Duration of study (week)	3	≤8 weeks	−0.86	−1.19, −0.53	0.0	
		1	>8 weeks	−1.05	−1.34, −0.76	-	
Adiponectin	Dosage of curcumin (mg/day)	2	≤500 (mg/day)	0.65	−0.11, 1.41	50.4	94.5
		3	>500 (mg/day)	1.28	0.15, 2.41	97.1	
	Duration of study (week)	3	≤8 weeks	0.95	0.27, 1.63	70.3	
		2	>8 weeks	1.23	−0.49, 2.94	98.5	

In sensitivity analysis, to assess the impact of one by one of all the included studies on the strength of relation between curcumin intake and body composition, the pooled SMD was summarized pre and post excluding each RCT from the meta-analysis. After excluding each study from the analyses, we observed no considerable difference between the pre- and post-sensitivity pooled SMD for BMI, BW, and HR (**Table 4**). But after omitting the data from the study of Panahi et al. (2017b) for WC, we found that there was significant impact between pre- (SMD −0.25; 95% CI, −0.44, −0.05) and post- (SMD −0.20; 95% CI, −0.42, 0.00) sensitivity pooled SMD for their body compositions.

**Table 4:** The association between curcumin supplementation and body composition using sensitivity analysis.

Variable	Pre-sensitivity analysis			Upper and lower of effect size	Post-sensitivity analysis		
	No. of studies included	Pooled SMD (random effect)	95% CI		Pooled SMD (random effect)	95% CI	Excluded studies
BMI	14	−0.37	−0.61, −0.13	Upper	−0.30	−0.51, −0.08	Panahi #2
				Lower	−0.41	−0.66, −0.17	Kocher
Body weight	15	−0.23	−0.39, −0.06	Upper	−0.17	−0.29, −0.05	Rahmani
				Lower	−0.25	−0.42, −0.08	Kocher
WC	9	−0.25	−0.44, −0.05	Upper	−0.17	−0.33, −0.01	Churngsamarn (2012)
				Lower	−0.20	−0.42, 0.00	Panahi #1
HR	5	−0.17	−0.42, 0.08	Upper	−0.02	−0.37, 0.31	Amin
				Lower	−0.20	−0.46, 0.06	Mohammadi (b)
Leptin	4	−0.97	−1.18, −0.75	Upper	−0.85	−1.18, −0.53	Churngsamarn (2014)
				Lower	−1.01	−1.26, −0.75	Panahi
Adiponectin	5	1.05	0.23, 1.87	Upper	1.26	0.51, 2.00	Churngsamarn (2012)
				Lower	0.95	−0.09, 2.01	Panahi

### Pooled Effects of Curcumin on Adipokines

**Figure 4:**
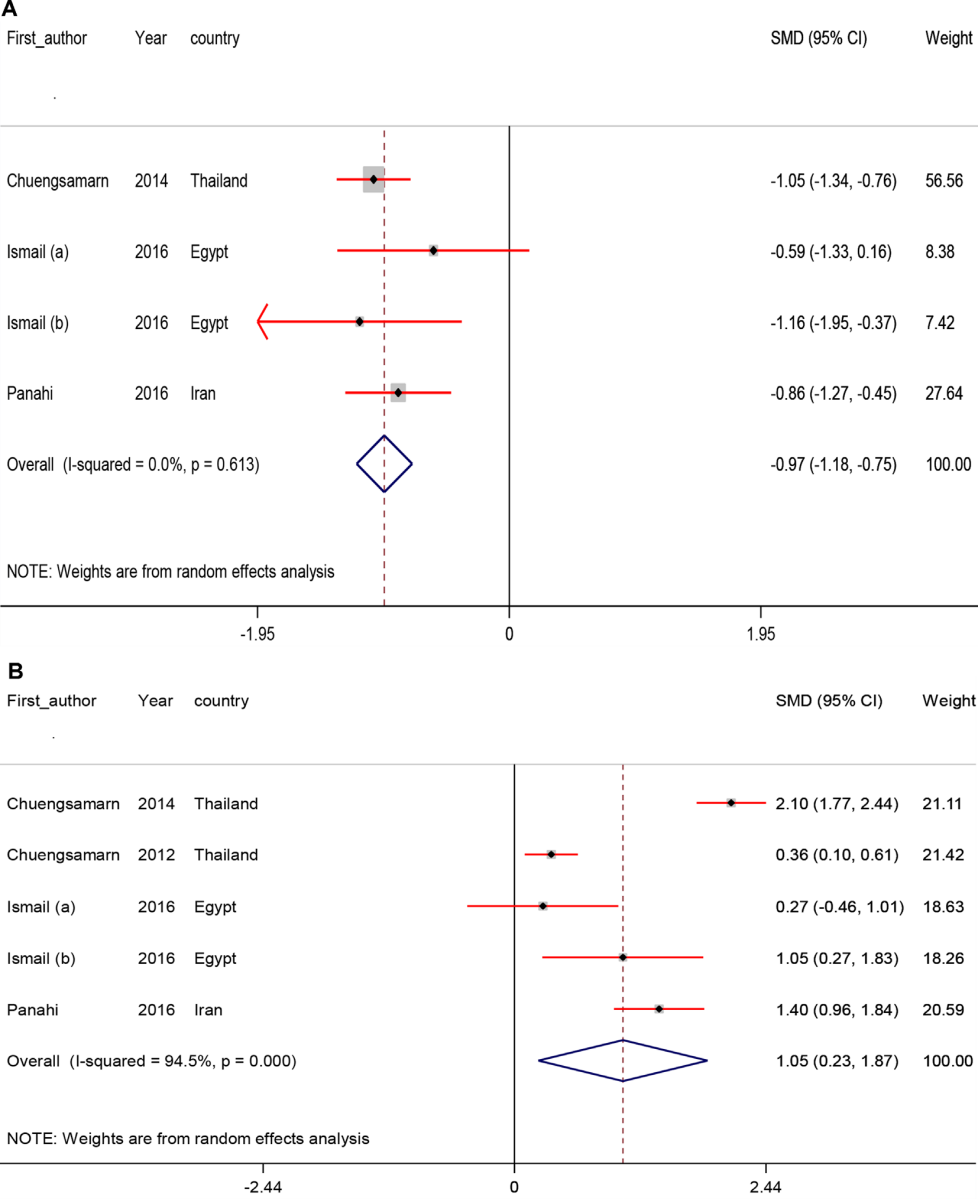
Meta-analysis adipokines standardized mean differences estimates for **(A)** leptin and for **(B)** adiponectin in curcumin supplements and placebo groups (CI = 95%).

The effects of curcumin intake on serum adipokines are presented in **Figure 4**. The results showed that curcumin intake among individuals with metabolic diseases significantly reduced leptin (SMD −0.97; 95% CI, −1.18, −0.75; *P* < 0.001) and increased adiponectin levels (SMD 1.05; 95% CI, 0.23, 1.87; *P* = 0.01).

According to findings of subgroup analyses, the reduction of heterogeneity was found in some of specific strata of suspected variables which the details of subgroup analyses were demonstrated in **Table 3**.

Sensitivity analysis was performed, and the results for serum leptin remained consistent with the pooled SMD. However, the lower and higher pooled SMD for serum leptin in the sensitivity analysis were −1.01 (95% CI: −1.26, −0.75) after omitting the data from Panahi et al. (2016) −0.85 (−1.18, −0.53) and after omitting the data from Chuengsamarn et al. (2014), respectively.

For serum adiponectin, we found that there was significant impact between pre- (SMD 1.05; 95% CI, 0.23, 1.87) and post- (SMD 0.95; 95% CI, −0.09, 2.01) sensitivity pooled SMD after omitting the data from the study of Panahi et al. (2016) (**Table 4**).

### Publication Bias

Egger’s regression test was applied to detect potential publication bias among the included studies in our meta-analyses. The findings of Egger’s regression test exhibited that no significant publication bias for meta-analyses estimating the effects of curcumin intake on BMI (*B* = 0.38, *P* = 0.84), BW (*B* = 0.28, *P* = 0.79), WC (*B* = 1.86, *P* = 0.11), serum leptin (*B* = 0.75, *P* = 0.56), and serum adiponectin (*B* = 1.14, *P* = 0.85).

But, there was evidence of the possible publication bias on the effects of curcumin intake on HR (*B* = 1.77, *P* < 0.01). Therefore, we used nonparametric method (Duval and Tweedie) to include the results of censored studies. The results indicated that summary effect sizes changed significantly for HR between before (SMD 0.17; 95% CI, −0.42, 0.08) and after (SMD −0.24; 95% CI, −0.46, −0.02) including the results of censored studies.

## Discussion

Our meta-analysis is the first report of the impact of curcumin intake on BMI, weight, body fat, leptin and adiponectin levels, and demonstrated that curcumin intake was correlated with a significantly decline in BMI, weight, WC, and leptin, and a significant increase in adiponectin levels, but did not affect HR.

MetS and obesity are associated with several metabolic and non-metabolic disorders such as CHD, T2DM, and certain types of cancer (Kromhout, 1983; Lynch et al., 2009). The current meta-analysis indicated that curcumin consumption resulted in a detectable reduction in BMI, weight, WC, and leptin, and a significant increase in adiponectin levels, but did not affect HR. Prior studies have evidenced that some spices may play a role in the management of obesity. In a meta-analysis by Hursel et al. (2011), it was seen that catechin-caffeine mixtures or a caffeine-only administration significantly induced energy expenditure. In addition, the intake of capsaicinoids decreased body weight through reductions in energy intake (Whiting et al., 2014). In another meta-analysis study, taking ginger by obese people decreased body weight, WC, HR, fasting glucose, and insulin resistance, and increased HDL-cholesterol, whereas did not influence insulin, BMI, triglycerides, total- and low-density lipoprotein (LDL)-cholesterol concentrations (Maharlouei et al., 2018). Moreover, catechins intake reduced weight as well as maintained weight after a period of weight loss (Hursel et al., 2009). On the other hand, some investigators have proved the useful effects of curcumin on the management of obesity. For example, a detrimental decline in BMI and liver fat status was seen after the consumption of 70 mg/day curcumin for 8 weeks in individuals with non-alcoholic fatty liver disease (NAFLD) (Rahmani et al., 2016). Taking nano-curcumin at a dose of 800 mg/day for 3 months by people with T2DM reduced BMI, hemoglobin A1c (HbA1c), fasting glucose, and triglycerides levels (Rahimi et al., 2016). However, some studies did not observe such useful effects of curcumin intake on BMI and body weight. For instance, anthropometric parameters such as BMI, weight, waist and hip circumference, and total body fat did not affect after the intake of curcumin at a dosage of 1 g/day in obese people (Mohammadi et al., 2013).

Inhibiting key transcriptional proteins related to adipogenesis may result in weight reduction and decreasing adipose tissue (Ejaz et al., 2009). In an animal study, suppressing angiogenesis in adipose tissue, downregulating preadipocyte differentiation, and upregulating adipocyte energy metabolism and apoptosis by curcumin intake led to lower body fat (Ejaz et al., 2009). Curcumin intake also up-regulates the gene expression of phosphorylated AMP-activated protein kinase (AMPK), perhaps *via* liver kinase B1, and this activated AMPK, which subsequently can function as a sirtuin 1 (SIRT1) activator (Binion et al., 2008). It would be predicted that activators of SIRT1 in adipose tissue would decrease inflammatory cytokine secretion, reduce macrophage infiltration, and improve insulin sensitivity (Yoshizaki et al., 2010). In addition, inhibiting pro-inflammatory cytokines such as tumor necrosis factor-α, plasminogen activator inhibitor type-1, and monocyte chemoattractant protein-1 following the intake of curcumin may justify its effect on weight loss (Bradford, 2013). Reducing BMI, weight, and total fat, and increasing percentage of lean mass are extremely useful toward increased insulin sensitivity and decreased risk of cardiovascular disturbances in obese individuals (Weisberg et al., 2008). Furthermore, curcumin down-regulates Janus Kinase enzyme which has been documented to have a fundamental function in obesity pathogenesis (Wang et al., 2009). Curcumin may also suppress 11beta-hydroxysteroid dehydrogenase enzyme which activates cortisol (Hu et al., 2013). Higher levels of cortisol in adipocytes induces central obesity (Kumari et al., 2010). In addition, curcumin reduces weight and BMI through the inhibition of adipocyte differentiation in the early stages *via* suppression of transcription factor peroxisome proliferator-activated receptor gamma (PPAR-γ) and by increasing monophosphate-activated protein kinase and consequently lipolysis (Lee et al., 2009). Curcumin may increase adiponectin expression in adipocyte through increased PPAR-γ expression (Dong et al., 2007). Activation of PPAR-γ results in enhanced insulin sensitivity in skeletal muscle and liver, and improves the secretory profiles of adipose tissue, favoring release of insulin-sensitizing adipokines including adiponectin, and decreasing inflammatory cytokines (Astapova and Leff, 2012). The inhibitory effects of curcumin on expression of estrogen receptor alpha (ERα) and the increasing of ERβ/Erα gene expression ratio also would result in a significant decrease in expression of both leptin and leptin receptor (Nejati-Koshki et al., 2014).

In view of the existing evidence, curcumin intake could be suggested as an effective supplement to be used for the management of MetS. A particular advantage of curcumin is their safety. Curcuminoids have been approved by US FDA as “generally recognized as safe” (GRAS), and their good tolerability and toxicity profile has been documented by several clinical trials (Sahebkar et al., 2013; Panahi et al., 2014; Mohammadi et al., 2017), even at high doses between 4,000 and 8,000 mg/day (Basnet and Skalko-Basnet, 2011).

### Limitations

The main limitation of the current study is that most of the included studies were performed with unformulated curcumin, which has low bioavailability due to poor absorption, rapid metabolism, and rapid removal from the body. Also, included studies had a low to moderate methodological quality. Furthermore, significant heterogeneity among included studies might be explained by the use of different methodologies, various dosages of curcumin used and in different durations, and evaluation of different populations. There were few eligible RCTs and a modest number of participants to be included in the meta-analysis to analyze the clinical effectiveness of curcumin on weight loss.

## Conclusions

Overall, the current meta-analysis demonstrated that curcumin intake significantly decreased BMI, weight, WC, and leptin, and significantly increased adiponectin levels, but did not affect HR.

## Author Contributions

ZA contributed in conception, design, statistical analysis and drafting of the manuscript. MA, KL, RT, MG-M, PP, GF and AG contributed in data collection and manuscript drafting. All authors approved the final version for submission. ZA supervised the study.

## Funding

The current study was founded by a grant number (97-01-106-16976) from the Vice-chancellor for Research, Shiraz University of Medical Sciences, Shiraz, Iran.

## Conflict of Interest Statement

The authors declare that the research was conducted in the absence of any commercial or financial relationships that could be construed as a potential conflict of interest.
